# Supplementary material for: Development of an RNA interference (RNAi) gene knockdown protocol in the anaerobic gut fungus Pecoramyces ruminantium strain C1A
Source: PeerJ. 2018 Jan 30;6:e4276. doi: 10.7717/peerj.4276 (PMC5796279; doi:10.7717/peerj.4276)
Supplement: Supplemental Information 1 — Figure S1. Uptake of fluorescently (Cy3) tagged siRNA by C1A spores. (A) The ldhD-specific siRNA was added to the flooding solution 75 minutes after the onset of flooding followed by incubation for 15 more minutes at 39°C. Samples (a few microliters) were taken at regular intervals for visualization. The same field is shown for DAPI-, and Cy3-labeled germinating spores (Note that the spores were concurrently stained with DAPI and fluorescing green indicating the uptake of the Cy3-labeled siRNA) (bar =2000B5). (B) Effect of the siRNA treatment on fungal growth rate. siRNA-treated spores were collected and used to inoculate fresh RFC medium. Control cultures were started at the same time using siRNA-untreated spores. Headspace pressure was measured daily and used to calculate fungal biomass as described previously (1). Error bars are standard deviations from at least three replicate cultures for each condition. Table S1. Silencing efficiency (based on the total number of mmoles of D-lactate in the culture supernatant) of ldhD-siRNA during early log (6–13 mg biomass), mid-log (14–17 mg biomass), and late log/early stationary (18–23 mg) phases a. Table S2. Transcripts with a significant (False Discovery Rate (FDR) <0.1) fold change in the ldhD-siRNA-treated cultures. [file peerj-06-4276-s001.docx]

Supplementary material for :

**Development of an RNA interference (RNAi) gene knockdown protocol in the anaerobic gut fungus *Pecoramyces ruminantium* strain C1A**

Shelby Calkins^1^, Nicole C. Elledge^1^, Stephen M. Marek^2^, MB Couger^3^, Mostafa S. Elshahed^1^, and Noha H. Youssef^1*^

^1^Department of Microbiology and Molecular Genetics, ^2^Department of Entomology and Plant Pathology, and ^3^High Performance Computing Center, Oklahoma State University, Stillwater, Oklahoma, USA

**Supplementary Tables.**

Table S1. Silencing efficiency (based on the total number of mmoles of D-lactate in the culture supernatant) of *ldhD*-siRNA during early log (6-13 mg biomass), mid-log (14-17 mg biomass), and late log/early stationary (18-23 mg) phases^a^.

| siRNA conc. (nM) | Fungal biomass (mg) | | |
| --- | --- | --- | --- |
|  | 6-13 mg | 14-17 mg | 18-23 mg |
| 20 | 58 | 51 | 43±28 |
| 50 | 42±30 |  | 41±5 |
| 75 | 14±7^*^ | 38±5^*^ | 32 |
| 100 | 28±3^#^ | 33±0.5^*^ | 14±2^*, #^ |

a: Silencing efficiency was calculated as follows: average total mmoles of D-lactate in culture supernatant in *ldhD*-siRNA treated cultures/ average total mmoles of D-lactate in culture supernatant in untreated cultures x 100. Standard deviation from at least two replicates is shown. Numbers with the same superscript symbol are significantly different (Student t-test p-value < 0.005).

Table S2. Transcripts with a significant (False Discovery Rate (FDR) < 0.1) fold change in the *ldhD*-siRNA-treated cultures.

| Transcript ID | IMG Gene accession number | Predicted function | Log_2_ fold change [TPM in siRNA-treated cultures/ TPM in untreated cultures] | -log_10_ FDR |
| --- | --- | --- | --- | --- |
| Significantly down-regulated transcripts | | | | |
| TRINITY_DN25404_c0_g1_i1 | 2511056874 | Actin and related proteins; ACTR8, ARP8, INO80N actin-related protein 8 | -9.89 | 3.03 |
| TRINITY_DN27647_c0_g2_i1 | 2511052130 | Ankyrin repeat | -8.89 | 1.22 |
| TRINITY_DN26914_c3_g1_i10 | 2511052359 | Aspartyl aminopeptidase | -11.31 | 1.04 |
| TRINITY_DN27318_c0_g1_i15 | 2511054689 | Beta-1,4-xylanase | -10.27 | 3.79 |
| TRINITY_DN27318_c0_g1_i3 | 2511054689 | Beta-1,4-xylanase | -9.47 | 2.21 |
| TRINITY_DN26767_c0_g1_i2 | 2518731831 | chloride channel 3/4/5 | -8.94 | 1.37 |
| TRINITY_DN27688_c5_g4_i3 | 2511053193 | DNA methylase | -9.97 | 3.24 |
| TRINITY_DN26958_c2_g1_i9 | 2511057393 | DnaJ family protein C member 3 | -8.83 | 1.22 |
| TRINITY_DN27149_c6_g1_i13 | 2511052023 | Endoglucanase | -11.50 | 1.13 |
| TRINITY_DN26597_c3_g6_i4 | 2511059612 | Endoglucanase | -9.65 | 2.58 |
| TRINITY_DN24006_c1_g2_i3 | 2511053004 | Fumarase | -9.13 | 1.34 |
| TRINITY_DN27371_c7_g1_i12 | 2511061203 | Glycosyl hydrolase family 9 | -9.31 | 1.47 |
| TRINITY_DN23827_c0_g1_i1 | 2511061779 | Hypothetical protein | -11.23 | 1.03 |
| TRINITY_DN22650_c0_g1_i1 | 2511057661 | Hypothetical protein | -11.21 | 1.08 |
| TRINITY_DN26878_c4_g5_i1 | 2518722908 | Hypothetical protein | -10.19 | 3.71 |
| TRINITY_DN72241_c0_g1_i1 | 2511055911 | Hypothetical protein | -9.79 | 2.88 |
| TRINITY_DN25954_c3_g3_i7 | 2511052693 | Hypothetical protein | -9.34 | 1.39 |
| TRINITY_DN27282_c5_g1_i4 | 2511054952 | Hypothetical protein | -9.32 | 1.70 |
| TRINITY_DN20666_c0_g1_i2 | 2511055315 | Hypothetical protein | -9.24 | 1.71 |
| TRINITY_DN24836_c0_g1_i3 | 2511055352 | Hypothetical protein | -9.10 | 1.57 |
| TRINITY_DN27173_c0_g2_i1 | 2511052832 | Hypothetical protein | -8.95 | 1.37 |
| TRINITY_DN26709_c2_g3_i8 | 2511053925 | Hypothetical protein | -8.83 | 1.18 |
| TRINITY_DN22602_c0_g1_i7 | 2511058339 | Hypothetical protein | -8.23 | 1.10 |
| TRINITY_DN61803_c0_g1_i2 | 2511052980 | Permeases of the drug/metabolite transporter (DMT) | -10.17 | 3.71 |
| TRINITY_DN25216_c1_g1_i1 | 2511061481 | Serine/threonine protein kinase | -8.74 | 1.10 |
| TRINITY_DN26990_c2_g2_i2 | 2511049217 | Uncharacterized conserved protein | -8.76 | 1.08 |
| TRINITY_DN23344_c0_g2_i1 | 2511048410 | V8-like Glu-specific endopeptidase | -9.42 | 1.67 |
| TRINITY_DN26795_c1_g3_i15 | 2511062537 | Vacuolar sorting protein 9 | -9.72 | 2.64 |
| TRINITY_DN27051_c5_g9_i6 | 2511059070 | WD40-repeat-containing domain | -9.17 | 1.55 |
| TRINITY_DN27737_c3_g9_i1 | 2511055262 | d-Lactate dehydrogenase | -1.31 | 1.02 |
|  | | | | |
| Significantly up-regulated transcripts | | | | |
| TRINITY_DN23455_c1_g3_i1 | 2511053442 | NAD-dependent 2-hydroxyacid dehydrogenase (Pfam00389) | 10.59 | 1.67 |
| TRINITY_DN26116_c0_g1_i2 | 2518732204 | ABC-type multidrug transport system, ATPase component | 9.03 | 1.08 |
| TRINITY_DN27116_c8_g3_i1 | 2518720443 | ABC-type multidrug transport system, ATPase component | 9.04 | 1.22 |
| TRINITY_DN25404_c0_g1_i6 | 2518716333 | Actin and related proteins; ACTR8, ARP8, INO80N actin-related protein 8 | 9.23 | 1.67 |
| TRINITY_DN25945_c5_g1_i1 | 2518724113 | Ankyrin repeat | 11.62 | 1.55 |
| TRINITY_DN27367_c4_g7_i1 | 2518717840 | Beta-ketoacyl synthase, N-terminal domain/AMP-binding | 9.08 | 1.53 |
| TRINITY_DN26659_c2_g1_i6 | 2518724693 | Calponin | 10.43 | 2.98 |
| TRINITY_DN27260_c3_g1_i2 | 2518717204 | Cell division protein | 9.28 | 1.85 |
| TRINITY_DN25040_c0_g1_i2 | 2518723847 | Aminopeptidase | 10.37 | 3.88 |
| TRINITY_DN84844_c0_g1_i1 | 2518724165 | Cyclin | 9.38 | 2.05 |
| TRINITY_DN27688_c5_g4_i4 | 2518731843 | Zinc finger, PHD-type | 8.13 | 3.79 |
| TRINITY_DN27658_c7_g1_i9 | 2518727414 | Enterochelin esterase and related enzymes | 9.41 | 1.38 |
| TRINITY_DN27456_c0_g1_i1 | 2518732213 | Transposase | 8.89 | 1.30 |
| TRINITY_DN27284_c8_g3_i8 | 2518722845 | Galactose binding lectin domain | 9.56 | 1.53 |
| TRINITY_DN27371_c7_g1_i8 | 2511050919 | Glycosyl hydrolase family 9 | 8.94 | 1.26 |
| TRINITY_DN27514_c12_g7_i3 | 2511058314 | Glycosyl hydrolase family 9 | 9.10 | 1.50 |
| TRINITY_DN26559_c2_g2_i3 | 2511061591 | Hypothetical protein | 4.35 | 1.04 |
| TRINITY_DN26871_c0_g1_i6 | 2511048726 | Hypothetical protein | 5.66 | 1.08 |
| TRINITY_DN26577_c7_g3_i6 | 2511060029 | Hypothetical protein | 7.06 | 3.71 |
| TRINITY_DN27338_c2_g6_i9 | 2511050279 | Hypothetical protein | 7.91 | 3.71 |
| TRINITY_DN20198_c0_g1_i1 | 2511054063 | Hypothetical protein | 8.64 | 1.04 |
| TRINITY_DN59842_c0_g2_i1 | 2511053000 | Hypothetical protein | 8.67 | 1.08 |
| TRINITY_DN26705_c9_g11_i5 | 2511061429 | Hypothetical protein | 8.79 | 1.10 |
| TRINITY_DN24643_c0_g3_i3 | 2511061911 | Hypothetical protein | 9.24 | 1.78 |
| TRINITY_DN26861_c6_g1_i1 | 2518722966 | Hypothetical protein | 9.36 | 1.68 |
| TRINITY_DN26789_c7_g2_i2 | 2511060353 | Hypothetical protein | 9.63 | 2.59 |
| TRINITY_DN25732_c3_g1_i2 | 2518716704 | CAP-Gly domain | 9.74 | 2.81 |
| TRINITY_DN20193_c0_g1_i1 | 2511049097 | Hypothetical protein | 9.74 | 2.27 |
| TRINITY_DN27291_c5_g1_i27 | 2511062706 | Hypothetical protein | 9.92 | 1.34 |
| TRINITY_DN26268_c0_g6_i2 | 2511056562 | Hypothetical protein | 9.94 | 3.10 |
| TRINITY_DN72241_c0_g4_i1 | 2511055911 | Hypothetical protein | 9.99 | 3.10 |
| TRINITY_DN23108_c0_g1_i1 | 2511055897 | Leucine-rich repeat (LRR) protein | 8.67 | 1.04 |
| TRINITY_DN26891_c1_g2_i3 | 2518723924 | Long-chain acyl-CoA synthetases (AMP-forming) | 9.22 | 1.59 |
| TRINITY_DN27245_c11_g4_i1 | 2511055785 | Metal-dependent hydrolase | 11.86 | 1.14 |
| TRINITY_DN26212_c0_g1_i6 | 2511051817 | Mismatch repair ATPase (MutS family) | 9.29 | 1.68 |
| TRINITY_DN26388_c1_g1_i3 | 2511051700 | Nucleosome-binding factor SPN, POB3 subunit | 8.96 | 1.26 |
| TRINITY_DN25459_c0_g1_i3 | 2518723044 | Nucleotide-sugar transporter. | 8.72 | 1.08 |
| TRINITY_DN26193_c4_g1_i3 | 2518729432 | Phosphatidylinositol-4-phosphate 5-Kinase | 9.92 | 3.10 |
| TRINITY_DN18878_c0_g1_i3 | 2511061142 | Predicted Rossmann fold nucleotide-binding protein | 12.17 | 9.52 |
| TRINITY_DN27738_c2_g1_i2 | 2518732026 | Reverse transcriptase (RNA-dependent DNA polymerase)/Integrase | 9.48 | 1.71 |
| TRINITY_DN26725_c0_g1_i2 | 2511052637 | RhoGEF domain. | 9.24 | 1.78 |
| TRINITY_DN25303_c1_g1_i1 | 2511056187 | Ribosomal protein L11 methylase | 9.49 | 1.90 |
| TRINITY_DN26960_c5_g2_i8 | 2518725849 | Superfamily II DNA/RNA helicases, SNF2 family | 9.01 | 1.35 |
| TRINITY_DN11227_c0_g2_i1 | 2511055978 | Trehalose-6-phosphate synthase | 12.34 | 1.26 |
| TRINITY_DN27057_c7_g3_i8 | 2511061879 | WD40-repeat-containing domain | 9.13 | 1.62 |
| TRINITY_DN24706_c0_g1_i2 | 2518725042 | WD40-repeat-containing domain | 9.31 | 1.55 |
| TRINITY_DN22853_c0_g1_i4 | 2518720070 | WD40-repeat-containing domain | 9.32 | 1.56 |
| TRINITY_DN27087_c6_g1_i1 | 2511051329 | WD40-repeat-containing domain | 9.36 | 1.84 |
| TRINITY_DN17665_c0_g1_i1 | 2518731672 | Zinc metalloprotease (elastase) | 9.56 | 2.22 |
| TRINITY_DN26802_c4_g9_i1 | NA^a^ | 1,4-alpha-glucan branching enzyme | 9.21 | 1.55 |
| TRINITY_DN27699_c2_g2_i5 | NA | Hypothetical protein | 9.65 | 2.62 |
| TRINITY_DN27163_c4_g2_i1 | NA | Hypothetical protein | 11.75 | 1.13 |
| TRINITY_DN19604_c0_g3_i1 | NA | SH3 domain | 8.89 | 1.26 |

a: NA Blastx comparison of the transcript sequence against C1A proteins showed no hits.

**Supplementary Figures**

Figure S1. Uptake of fluorescently (Cy3) tagged siRNA by C1A spores. (A) The *ldhD*-specific siRNA was added to the flooding solution 75 minutes after the onset of flooding followed by incubation for 15 more minutes at 39ºC. Samples (a few microliters) were taken at regular intervals for visualization. The same field is shown for DAPI-, and Cy3-labeled germinating spores (Note that the spores were concurrently stained with DAPI and fluorescing green indicating the uptake of the Cy3-labeled siRNA) (bar=20 µm). (B) Effect of the siRNA treatment on fungal growth rate. siRNA-treated spores were collected and used to inoculate fresh RFC medium. Control cultures were started at the same time using siRNA-untreated spores. Headspace pressure was measured daily and used to calculate fungal biomass as described previously (1). Error bars are standard deviations from at least three replicate cultures for each condition.

**References:**

1. **Ranganathan A, Smith OP, Youssef NH, Struchtemeyer CG, Atiyeh HK, Elshahed MS.** 2017. Utilizing anaerobic fungi for two-stage sugar extraction and biofuel production from lignocellulosic biomass. Front Microbiol **8:**635.
